# Skeletal Muscle Mass and Explosive Strength in Young, Healthy Adults: Associations with Gait Biomechanics, Speed and Sedentary Behavior—A Cross-Sectional Study

**DOI:** 10.3390/jfmk11040404

**Published:** 2026-10-03

**Authors:** Dolores Hinić, Ivan Veber, Dunja Igrec, Katarina Jantoš, Anita Matić

**Affiliations:** 1 Faculty of Dental Medicine and Health Osijek, Josip Juraj Strossmayer University in Osijek, Crkvena 21, 31 000 Osijek, Croatia; dhinic@fdmz.hr (D.H.); kjantos@fdmz.hr (K.J.); 2 Department of Integrative Medicine, Faculty of Dental Medicine and Health Osijek, Josip Juraj Strossmayer University in Osijek, Crkvena 21, 31 000 Osijek, Croatia; iveber@fdmz.hr; 3 Department of Pathophysiology, Physiology and Immunology, Faculty of Dental Medicine and Health Osijek, Josip Juraj Strossmayer University in Osijek, Crkvena 21, 31 000 Osijek, Croatia; digrec@fdmz.hr

**Keywords:** sedentary behavior, physical activity, skeletal muscle mass, gait analysis, explosive strength, body composition, young adults, IPAQ

## Abstract

**Background and objectives:** Sedentary behavior and physical activity are important determinants of musculoskeletal health, yet their relationship with gait biomechanics, explosive strength, and speed remains understudied in healthy, young populations relative to older and clinical groups. This study primarily aimed to examine the relationship between skeletal muscle mass and explosive strength in a healthy, young population, while secondarily exploring its association with sedentary behavior, physical activity, and gait biomechanical parameters. **Methods:** A cross-sectional study included 37 healthy young adults. Physical activity and sedentary behavior were assessed using the long-form IPAQ, body composition using bioelectrical impedance analysis, and gait, explosive strength, and speed using a wearable inertial sensor system (countermovement and plyometric jumps, parkour test). Muscle mass–strength associations were also examined with partial correlation adjusted for sex. **Results:** Skeletal muscle mass percentage was positively associated with countermovement jump height and power and plyometric jump height, but these associations did not remain significant after adjustment for sex. Muscle mass was not associated with total IPAQ score and was only nominally associated with IPAQ activity category, an association that did not survive correction for multiple testing. Physical activity was not associated with any gait parameter, nor did gait asymmetry differ by activity category. Sedentary behavior was not associated with any gait, strength, or speed parameter, though men reported more sitting time than women, a difference that did not remain significant after correction for multiple testing. **Conclusions:** In healthy young adults, the unadjusted muscle mass–strength association largely reflected shared sex differences rather than an independent relationship, underscoring the importance of accounting for sex in this population. These preliminary findings support combining questionnaire-based, body-composition, and instrumented biomechanical assessment, with statistical adjustment, in future, larger, sex-balanced research.

## 1. Introduction

Sedentary behavior is characterised by an energy expenditure of ≤1.5 metabolic equivalents (METs) during waking activities such as sitting, reclining, or lying [1,2]. It should be distinguished from physical inactivity, since it can be high even alongside a moderate-to-high overall activity level [3,4]. Prolonged sitting increases the risk of cardiovascular disease, cancer, type 2 diabetes, obesity, and mental illnesses such as anxiety and depression [5,6,7,8,9]. Adults are generally assumed to spend more than half of their day seated [3], and recent German data show average weekday sitting time rising from 457 to 533 min in just nine years, with the proportion sitting over 8 h/day increasing from 42% to 55% [10]. Students show particularly pronounced sedentary behavior, spending up to 10 h a day at a computer, a figure that has risen with greater access to information and new learning modes [11]. This varies further by sex and education level [12], underscoring the need for further research into the factors driving sedentary behavior in this group [13].

Because prolonged sitting reduces the mechanical stimulus needed to maintain muscle mass, physical activity is an important consideration for improving health and quality of life at all ages [14]. Consistent physical activity, especially weight training along with aerobic exercise, significantly influences the growth and preservation of muscle mass [15,16], although it is still uncertain which training approach is the most effective; a mix of light, moderate, and heavy weights over training phases seems to be the most advantageous [17,18]. Muscle mass growth also varies by sex, with women generally advancing more slowly based on age, personal traits, fitness level, and nutrition [19], and more studies are necessary to understand differences in how populations respond to physical activity [17]. Because muscle mass plays a key role in generating and transmitting force during movement, it may also influence walking biomechanical patterns.

Walking is a fundamental mode of human locomotion, including a swing phase (about 40% of the cycle) and a stance phase (about 60%) [20,21,22], controlled by the foot, muscles, fascia, tendons, joints, and ligaments of the lower body [23] under nervous system guidance [24]. Gait analysis, particularly walking speed, strongly indicates functional status across age groups and sexes [25,26]. Standardized, multimodal methods (spatiotemporal, kinetic, electromyographic), including 3D analysis and wearable sensors, offer greater objectivity and precision in clinical and sports-related applications [27,28,29].

Spatiotemporal gait parameters, including cycle duration, stance and swing phases, step length, and asymmetry, are important indicators of functional status, with changes such as reduced gait speed and prolonged stance time described in clinical populations and with aging [30,31,32,33]. Everyday conditions can also affect gait in young, healthy individuals; for instance, using a mobile device while walking reduces cadence and step length [34]. Muscle mass affects gait parameters mainly in older adults, with limited effect on young, healthy individuals. However, greater quadriceps mass may shorten double- and single-limb support time, while greater total body mass impairs gait to a greater extent [35,36,37]. Alongside these absolute values, symmetry between the two legs is an additional, independent indicator of gait quality.

Symmetry between the two legs is a hallmark of healthy walking, and minor, functional deviations related to leg dominance are normal; asymmetry becomes clinically significant when more pronounced, as in neurological conditions [38]. Values up to 10% are generally considered normal, while asymmetry above 15% is considered clinically important [39]. In healthy individuals, asymmetry also varies with activity level: professional runners, for example, show better stability and less asymmetry than less active individuals [40,41,42]. However, most research on gait asymmetry has focused on older or clinical populations, leaving this relationship considerably less explored in young, healthy individuals, for whom asymmetry, explosive strength, and speed together shape overall functional capacity.

Explosive strength, the ability to rapidly produce maximal force, is a key component of athletic performance in sports involving fast, intense movements such as sprinting, jumping, and combat sports [43,44]. Speed also depends on nervous system function, contraction speed, and movement technique [45]. Plyometric training effectively develops explosive strength and sprint speed in both sexes, with men typically achieving better results due to a higher percentage of muscle and total body mass [46,47,48].

Although researchers have extensively studied sedentary behavior, physical exercise, muscle size, walking mechanics, and explosive strength and speed, most earlier studies have focused on older or clinical groups, where these elements are primarily examined in relation to reduced function. In particular, the relationship between skeletal muscle mass and explosive strength, though well established in older adults and clinical populations, remains comparatively unexamined in healthy young adults, who typically show preserved physical function and may therefore reveal this relationship independent of age- or disease-related decline. In contrast, few studies in a healthy, young group have combined questionnaire-based evaluations of sedentary behavior and physical exercise, objective bioimpedance analysis of body composition, and instrumented assessments of walking, strength, and speed within the same group. A physiotherapy student group is especially suitable for this method, combining significant academically related sedentary behavior with maintained physical function, free from the confounding effects of illness or aging. Building on this rationale, the present study primarily aimed to examine the relationship between skeletal muscle mass and explosive strength in a healthy, young population, while secondarily exploring its association with sedentary behavior, physical activity, and gait biomechanical parameters.

## 2. Materials and Methods

### 2.1. Study Design and Participants

A cross-sectional study was conducted. A total of 40 physiotherapy students, both sexes, from the Faculty of Dental Medicine and Health, Osijek, were recruited using a convenience sampling approach, and all agreed to participate. The study was conducted between March and June 2026. Prior to testing, a qualified physiotherapist conducted a standard subjective assessment to determine eligibility. The assessment included: history of current and past musculoskeletal injuries (especially of the lower limbs), surgeries, and pain; history of cardiovascular, neurological, and metabolic conditions; current medication use; self-reported functional limitations in walking, running, jumping, and stair climbing; and a brief examination of bilateral symmetry and the functional status of all limbs, joints, and major muscle groups. Participants reporting a condition that could influence test performance or compromise safety were excluded from the study. Three participants were excluded from the final analysis because they provided incomplete responses on the IPAQ (fewer than one third of the 25 detailed activity items were answered), resulting in unreliable physical activity scores; these participants completed all other testing procedures.

Sample size was estimated for the primary hypothesis (association between skeletal muscle mass and explosive strength), based on correlation coefficients reported in previous studies (*r* = 0.43–0.69) [49,50]. At the lower, more conservative end of this range (*r* = 0.45), a minimum of 37 participants was required to detect the association with 80% power at *α* = 0.05; at *r* = 0.50, a minimum of 29 participants was required. The present sample of 37 participants therefore meets the more conservative estimate. Ethical approval for the study was granted by the Ethics Committee of the Faculty of Dental Medicine and Health, Osijek, Croatia (Class: 602-01/26-12/07; Reference number: 2158/97-97-10-26-21).

### 2.2. Instruments and Procedures

Physical activity level and sedentary behavior were assessed using the long form of the International Physical Activity Questionnaire (IPAQ). In 27 questions, participants recalled the time spent on specific activities over the previous seven days, including activity at work, transport-related activity, household and family-care activities, recreation and leisure-time physical activity, and time spent sitting. The questionnaire is freely available under a CC BY 4.0 license [51]. Before completing the IPAQ, participants completed a short questionnaire on basic demographic characteristics, including sex, age, place of residence, and participation in organized sports activity. IPAQ scores were calculated by multiplying the reported frequency (days/week) and duration (minutes/day) of activity within each domain (work, transport, domestic and garden, and leisure-time) by the corresponding MET value (walking = 3.3, moderate activity = 4.0, vigorous activity = 8.0) and summing these products across domains to obtain total weekly MET-minutes. Reported activity durations exceeding 180 min/day were capped at 180 min/day prior to calculation, consistent with standard IPAQ data-cleaning procedures. Participants were categorized into low (<600 MET-min/week), moderate (600–2999 MET-min/week), or high (≥3000 MET-min/week) physical activity categories based on total weekly MET-minutes.

Body composition was assessed using a bioelectrical impedance device (InBody 270S, InBody Co., Ltd., Seoul, Republic of Korea), which measures body mass, total body water, body mass index, and skeletal muscle mass through the flow of a weak electrical current at three frequencies (20 kHz, 50 kHz, and 100 kHz) across the body’s five segments (right arm, left arm, trunk, right leg, left leg); each measurement lasted 30 s. For this study, the device was used to determine the percentage of skeletal muscle mass. Measurements were conducted in the morning, and participants were instructed not to exercise before testing. Prior to measurement, participants self-reported that they were not dehydrated, that a minimum of two hours had elapsed since their last meal, and that they had not consumed alcohol; participants also voided their bladder immediately before testing.

Gait biomechanical parameters, explosive strength, and speed were assessed using a wearable inertial sensor system (Euleria Lab, Euleria Health, Rovereto, Italy). Before each measurement, a participant profile was created on a dedicated laptop, including name, age, sex, height, body mass, side dominance, and any relevant health conditions. For gait analysis, two motion sensors were used: one placed on the dorsal side of the right foot and one on the dorsal side of the left foot, each secured with an accompanying strap to ensure stability during the test; following sensor placement and activation, the system was recalibrated before each measurement. Participants then walked at their self-selected pace for approximately 30 s while the device recorded spatiotemporal gait parameters. No fixed walkway length was used; participants completed 10 consecutive steps before turning and continuing in the opposite direction, repeating this pattern throughout the test so the number of turns and the length of each straight segment varied with each participant’s natural stride. The system automatically processed data collected during turns and straight-walking segments. The Euleria Lab software automatically derived gait speed, gait distance, stride length, step length, and spatial and temporal asymmetry indices (SAI and TAI) using an internal step-detection and gait-event algorithm; the manufacturer did not disclose the algorithm’s specific computational details.

Explosive strength was then assessed through countermovement jumps on the left leg, right leg, and both legs, as well as plyometric jumps, with one sensor placed at the mid-trunk (below the ribcage) and one on the mid-right shank, and jump height during ground contact was recorded. Speed was assessed using a parkour test, in which participants completed a hopscotch-like obstacle course as quickly as possible using one leg; completion time was recorded separately for each leg and used by the software to compute left–right speed asymmetry, which was the parkour outcome analyzed in this study (Supplementary Material Figure S1). Single-leg countermovement jump asymmetry and parkour asymmetry were likewise computed automatically using the manufacturer’s proprietary, undisclosed algorithm (Figure 1).

### 2.3. Statistical Analysis

Statistical analysis was performed on data collected through the questionnaire and measurements of biomechanical and motor parameters. Sociodemographic variables (sex, place of residence, and participation in organized sports activity) were analyzed as categorical variables and are presented as absolute (*N*) and relative frequencies (%). Age, anthropometric indicators, physical activity indicators, sedentary behavior, gait biomechanical parameters, explosive strength, and speed were analyzed as continuous variables and are presented as mean (*M*), standard deviation (*SD*), and minimum and maximum values.

Given the sample size and the distribution of individual variables, the Mann–Whitney *U* test was used to examine differences in sedentary behavior by sex, and the Kruskal–Wallis test was used to analyze differences in spatial and temporal gait asymmetry across IPAQ physical activity categories. Associations between percentage of body skeletal muscle mass, physical activity level, sedentary behavior, gait biomechanical parameters, explosive strength, and speed were analyzed using Spearman’s correlation coefficient. Ninety-five percent confidence intervals for Spearman correlation coefficients were calculated using Fisher’s *z*-transformation. Effect sizes were calculated alongside Mann–Whitney *U* and Kruskal–Wallis test results (*r* for Mann–Whitney; epsilon-squared [ε^2^] for Kruskal–Wallis). For variables with skewed distributions, median and interquartile range (IQR) were additionally reported.

Given the number of correlational and group comparisons performed, *p*-values were additionally adjusted for multiple testing using the Benjamini–Hochberg false discovery rate (FDR) procedure, applied across all 24 hypothesis tests reported in this study; both unadjusted and FDR-adjusted *p*-values are reported. To address the potential confounding effect of sex on the association between skeletal muscle mass and explosive strength, partial Spearman correlation analyses adjusted for sex were additionally performed for these associations. These sex-adjusted partial correlations were treated as sensitivity analyses and were not included in the FDR family.

Statistical significance for all tests was set at *p* < 0.05. Statistical analysis was performed using IBM SPSS Statistics, version 29.0.2.0. (IBM Corp., Armonk, NY, USA).

## 3. Results

### 3.1. Sociodemographic and Anthropometric Characteristics of Participants

A total of 37 participants were included. The sample included 27 women (73.0%) and 10 men (27.0%). By residence, 22 participants (59.5%) lived in a city, and 15 (40.5%) lived in a rural area. Regarding organized sports participation, 21 participants (56.8%) did not participate, 11 (29.7%) participated recreationally, and 5 (13.5%) participated competitively (Table 1). By IPAQ categorization, most participants were in the high physical activity category (29 participants, 78.4%), followed by the moderate physical activity category (6 participants, 16.2%). In comparison, 2 participants (5.4%) belonged to the low physical activity category (Table 1).

### 3.2. Sedentary Behavior

Daily sitting time was calculated as the weighted average of self-reported weekday and weekend sitting time: (weekday minutes × 5 + weekend minutes × 2)/7. Sitting-time data were unavailable for 4 of 37 participants (10.8%) due to non-numeric or unparseable free-text responses to the weekday and/or weekend sitting-time items (e.g., a response given in days rather than duration, or a verbal description without a specific numeric value), resulting in a sitting-time sample of 33 participants. Men reported longer daily sitting time (Median = 7.71, IQR = 5.50–8.79; *M* = 7.16, *SD* = 2.01, *N* = 8) than women (Median = 5.00, IQR = 3.86–6.25; *M* = 5.36, *SD* = 2.12, *N* = 25), Mann–Whitney *U* = 151.00, *p* = 0.034, *r* = 0.37, 95% CI (0.07, 0.64) (medium effect); however, this difference did not remain statistically significant after FDR correction (*p* (FDR) = 0.192) (Table 2).

### 3.3. Descriptive Statistics of the Main Variables

Descriptive analysis of anthropometric indicators, physical activity level, sedentary behavior, gait biomechanical parameters, explosive strength, and speed was performed to provide a more detailed overview of the main study variables. Results are presented in Table 3. Because of missing data for individual measurement instruments, the number of participants included varied across variables (*N* = 33–37), as shown in Table 3. On average, participants had a normal body mass index, while the percentage of skeletal muscle mass varied more across the sample. Considerable dispersion was also evident in total physical activity.

### 3.4. Association Between Percentage of Skeletal Muscle Mass and Physical Activity Level

Spearman correlation analysis was used to examine the association between percentage of body skeletal muscle mass and physical activity level. No statistically significant association was found between percentage of skeletal muscle mass and total IPAQ score expressed in MET-min/week (*ρ* = 0.224; 95% CI (−0.11, 0.51); *p* = 0.183; *p* (FDR) = 0.430). A nominally significant positive association was found between percentage of skeletal muscle mass and IPAQ physical activity category (*ρ* = 0.339; 95% CI (0.02, 0.60); *p* = 0.040); however, this association did not remain statistically significant after FDR correction (*p* (FDR) = 0.192) (Table 4).

### 3.5. Association Between Physical Activity Level and Gait Biomechanical Parameters

No statistically significant associations were found between total physical activity level and any gait biomechanical parameter (Table 5).

### 3.6. Association Between Percentage of Skeletal Muscle Mass and Lower-Limb Explosive Strength, Before and After Adjustment for Sex

A higher percentage of skeletal muscle mass was associated with better performance on explosive strength tests, specifically CMJ height (*ρ* = 0.481; 95% CI (0.18, 0.70); *p* = 0.003), CMJ power (*ρ* = 0.569; 95% CI (0.30, 0.76); *p* < 0.001), and plyometric jump height (*ρ* = 0.503; 95% CI (0.21, 0.71); *p* = 0.002), and these unadjusted associations remained statistically significant after FDR correction (*p* (FDR) = 0.012 for CMJ power and 0.024 for CMJ height and plyometric jump height). However, none of these associations remained statistically significant after adjustment for sex (partial *ρ* = 0.092, 0.307, and 0.040, respectively; all *p* > 0.05), and the 95% confidence intervals for the sex-adjusted partial correlations crossed zero in all cases. No statistically significant association was found with indicators of lateral asymmetry (single-leg CMJ asymmetry and parkour asymmetry), either before or after adjustment (Table 6).

### 3.7. Spatial and Temporal Gait Asymmetry in Relation to Physical Activity Level

Neither temporal nor spatial gait asymmetry differed significantly by IPAQ category (TAI: *p* = 0.072, *p* (FDR) = 0.288, ε^2^ = 0.096; SAI: *p* = 0.983, *p* (FDR) = 0.983, ε^2^ = −0.058), although this comparison should be interpreted with caution given the very small low-activity subgroup (*N* = 2) (Table 7).

### 3.8. Association Between Percentage of Skeletal Muscle Mass and Lateral Speed Asymmetry (Parkour Test)

Spearman correlation analysis was used to examine the association between percentage of body skeletal muscle mass and the degree of lateral speed asymmetry between the left and right leg. No statistically significant association was found (*ρ* = 0.018; 95% CI (−0.32, 0.35); *p* = 0.920) (Table 8).

### 3.9. Association Between Sedentary Behavior and Biomechanical and Motor Parameters

No statistically significant associations were found between sedentary behavior and any gait, explosive strength, or speed parameter examined (Table 9).

## 4. Discussion

This study primarily examined the relationship between skeletal muscle mass and explosive strength in a healthy young population, while secondarily exploring its association with sedentary behavior, physical activity, and gait biomechanical parameters. The main results show that percentage of body skeletal muscle mass was associated with indicators of lower-limb explosive strength—specifically CMJ height, CMJ power, and plyometric jump height—although these associations did not remain statistically significant after adjustment for sex, indicating that they largely reflect sex differences in both skeletal muscle mass and explosive strength rather than an independent relationship; no association was found with indicators of lateral asymmetry (single-leg CMJ, parkour), either before or after adjustment. Percentage of skeletal muscle mass was not significantly associated with total IPAQ score, and was only nominally associated with IPAQ physical activity category, an association that did not remain significant after FDR correction. Physical activity level was not significantly associated with any of the examined gait biomechanical parameters, and neither temporal nor spatial gait asymmetry differed significantly across IPAQ categories. However, a non-significant descriptive pattern of greater temporal asymmetry in the low-activity subgroup should be regarded as exploratory given its very small size (*N* = 2). Sedentary behavior, expressed as average daily sitting time, was not associated with any biomechanical or motor parameters examined. Men, on average, reported more sitting time than women, although this difference did not remain significant after FDR correction.

The study included 37 physiotherapy students from the Faculty of Dental Medicine and Health Osijek, Croatia (Table 1), most of whom reported not participating in organized sport. Yet, a large majority nonetheless fell into the high physical activity category on the IPAQ (Table 1). High IPAQ scores may therefore reflect activity accumulated through domains such as transport, daily walking or cycling, household tasks, or work-related movement, rather than necessarily indicating additional structured exercise or participation in organized sport. This aligns with a 2022 systematic review reporting that university students generally show a significant level of physical activity and physical fitness, although results may vary by cultural and educational context [52]. The mean and median IPAQ total scores observed in this sample (*M* = 7761.06, Median = 6850.00 MET-min/week; Table 3) were considerably higher than those reported in earlier studies of student populations, typically in the range of 1300–3300 MET-min/week, though notably closer to more recent findings. Leśniak et al. [53], using the same IPAQ long form and identical data-cleaning procedures, reported mean total physical activity of 6224.03 and 6038.73 MET-min/week among Polish women and men medical and health-science students, respectively. The divergence between the mean and median values observed here reflects a right-skewed distribution, with a small number of highly active participants raising the mean above the more representative median; this pattern, together with the elevated overall activity level, may partly reflect genuine high activity among physiotherapy students, as well as the well-documented tendency of self-reported IPAQ data to overestimate activity relative to objective measures. Sedentary behavior, assessed as average daily sitting time (Table 3), was also higher among men than women, a difference in medium effect size (*r* = 0.37), although it did not remain statistically significant after FDR correction (*p* (FDR) = 0.192) (Table 2). This is consistent with previous reports that students spend much of their sedentary time studying or using electronic devices [54]. Possible explanations for this sex difference include differences in sample composition, device use, or transport habits.

After presenting the sample’s general characteristics and physical activity level, the analysis examined whether a direct relationship exists between body composition and participants’ activity level. The relationship between percentage of body skeletal muscle mass and physical activity was analyzed. No statistically significant evidence of an association was found between percentage of body skeletal muscle mass and the IPAQ score expressed in MET-min/week (*ρ* = 0.224; 95% CI (−0.11, 0.51); *p* = 0.183). In contrast, a nominally significant positive association was found between IPAQ physical activity category and percentage of body skeletal muscle mass (*ρ* = 0.339; 95% CI (0.02, 0.60); *p* = 0.040) (Table 4); however, this association did not remain statistically significant after FDR correction (*p* (FDR) = 0.192), and the confidence interval’s lower bound lies close to zero, indicating that it should be interpreted with caution. This divergence between the continuous and categorical IPAQ measures suggests that the relationship, if present, may be modest and sensitive to how physical activity is operationalized. These results can be compared with other studies. Štefan et al. [55] observed a positive association between physical activity and muscle mass in healthy young individuals, though cautioning that a cross-sectional design cannot establish causality; similarly, other work links physical activity or sport participation with higher muscle mass, aided by prior training [56]. However, Rojer et al. [57] found that in young individuals, physical activity is more strongly associated with muscle strength than with muscle mass itself, a finding partly consistent with the divergent pattern observed here. These mixed results may reflect the self-report nature of the IPAQ, individual variability in estimating activity intensity, and the influence of factors such as age, diet, and genetics on skeletal muscle mass independent of activity level. Longitudinal studies with larger, activity-characterized samples would help clarify this relationship.

Physical activity level, although assessed here through a subjective, questionnaire-based measure integrating habitual behavior across multiple domains, is generally expected to promote neuromuscular adaptations that could, in principle, be reflected in objectively measured motor outcomes such as gait biomechanics, explosive strength, and inter-limb symmetry; the following analyses therefore examined whether this expected link was observable in the present sample. Since the absence of a direct association between skeletal muscle mass and physical activity level does not rule out its possible role in movement itself, the association between physical activity level and gait biomechanical parameters was examined next. Spearman correlation analysis found no statistically significant evidence of an association between the physical activity component of the IPAQ and any of the examined gait parameters (all 95% confidence intervals crossed zero, Table 5), possibly reflecting the relatively homogeneous, physically active nature of this young sample, as well as limitations of the IPAQ, which does not distinguish activity type or intensity. This partly diverges from previous studies: physically active and inactive young individuals have been shown to use different gait patterns [58], and physically active young men have shown greater gait speed than inactive counterparts [59]. Similar associations reported in older populations, such as lower gait variability among active older adults [60], may reflect activity-related differences that are more pronounced with age or with larger contrasts in activity intensity than observed in this predominantly active young sample; gait parameters here may instead be more strongly shaped by body build, motor ability, and individual gait characteristics than by physical activity level per se.

In contrast to the lack of association with overall physical activity level, skeletal muscle mass showed an association with direct indicators of muscle function that warranted closer examination; we therefore next examined the relationship between percentage of body skeletal muscle mass and lower-limb explosive strength. Unadjusted analysis showed a statistically significant positive association between percentage of body skeletal muscle mass and two-legged jump height (CMJ height), two-legged jump power, and plyometric jump height, which remained significant after FDR correction (*p* (FDR) = 0.012 for CMJ power and 0.024 for CMJ height and plyometric jump height) (Table 6). However, because both skeletal muscle mass and explosive strength are strongly related to sex, and because the sample was predominantly female, we re-examined these associations using partial Spearman correlation adjusted for sex. None of the three associations remained statistically significant after adjustment, and the 95% confidence intervals for the sex-adjusted partial correlations crossed zero in all cases. This indicates that the unadjusted associations largely, and in some cases fully, reflect the shared relationship of skeletal muscle mass and explosive strength with sex, rather than an association between the two variables that is independent of sex. No statistically significant evidence of an association was found between skeletal muscle mass and single-leg CMJ asymmetry (*ρ* = 0.059; 95% CI (−0.29, 0.39)) or parkour asymmetry (*ρ* = 0.018; 95% CI (−0.32, 0.35)), either before or after adjustment.

These findings should be interpreted with corresponding caution. Rather than indicating that greater skeletal muscle mass itself explains better jumping performance, the results are more consistent with skeletal muscle mass and explosive strength representing parallel, sex-related characteristics in this sample. Several other factors may also contribute to explosive strength independently of skeletal muscle mass, particularly training level, especially when training is focused on strength and explosiveness, as well as jump execution technique or neuromuscular control. The lack of an association between skeletal muscle mass and asymmetry in left–right jumps and parkour performance may reflect that these parameters depend more on limb dominance, coordination, and the mechanical properties of the locomotor system than on skeletal muscle mass itself. This is consistent with a growing body of recent literature indicating that inter-limb asymmetry is task-, metric-, and individual-dependent and does not necessarily parallel bilateral performance. Papale et al. [61], for instance, found that inter-limb asymmetry in young healthy adults responded differently from bilateral CMJ performance following an acute exercise stimulus, with asymmetry improving in participants with higher baseline asymmetry while CMJ height remained largely unchanged. A systematic review and a narrative review have also highlighted substantial methodological variability in how asymmetry is calculated and reported, along with an inconsistent relationship between asymmetry magnitude and physical performance [62,63]. Taken together, this literature suggests that the absence of an association between muscle mass and asymmetry observed in the present study should not be interpreted as unexpected, but rather as consistent with the broader understanding that bilateral strength or muscle-mass measures and inter-limb asymmetry capture largely independent aspects of neuromuscular function.

Comparing these results with previous studies reveals both similarities and an important methodological distinction. Correas-Gómez et al. [64] observed that greater lean body mass, including bone, water, organs, blood, and other tissues, is associated with better explosive strength in the two-legged vertical jump in young individuals; similarly, Maden-Wilkinson et al. [50] report that muscle mass, particularly in the quadriceps, is associated with explosive strength through increased maximal force-generating capacity, while also noting the contribution of neurological factors to rapid force development. Neither study, however, reports analyses adjusted for sex, and their samples, like the unadjusted results of the present study, may be similarly affected by this potential confounder. More longitudinal, sex-stratified studies that track changes within each sex are needed to more precisely establish whether a genuine, sex-independent relationship exists between increased skeletal muscle mass and better performance on explosive strength tests, as well as its association with left–right asymmetry.

Beyond absolute indicators of explosive strength, whether a difference exists in gait symmetry itself according to physical activity level was additionally examined. The relationship between spatial and temporal gait asymmetry and physical activity level, assessed by the IPAQ, was analyzed next. Spatial and temporal gait asymmetry did not differ significantly across IPAQ physical activity categories (Table 7), effect sizes were negligible for spatial asymmetry (ε^2^ = −0.058) and moderate for temporal asymmetry (ε^2^ = 0.096), although the latter estimate is imprecise given the very small low-activity subgroup. A descriptive pattern was nonetheless observed, with participants in the low physical activity category showing numerically higher temporal asymmetry values than those in the moderate or high categories; however, given the very small size of the low-activity group (*N* = 2), the absence of post hoc pairwise comparisons, and the number of hypothesis tests performed across this study, this pattern should be regarded as exploratory and hypothesis-generating only, rather than evidence that low physical activity is associated with impaired temporal gait symmetry. Any interpretation of this descriptive pattern, including possible links to neuromuscular control, leg dominance coordination, or individual gait characteristics, should accordingly be treated as speculative pending confirmation in larger, more balanced samples.

The relationship between physical activity and temporal and spatial gait parameters has not been sufficiently studied, particularly in young individuals, which limits how directly the present, nonsignificant findings can be compared with prior work. Some studies do report associations between physical activity and gait characteristics that may nonetheless inform future hypotheses in this area. Lee et al. [65] analyzed gait in young, healthy individuals and identified spatial and temporal parameters, along with bilateral coordination, as important components of gait analysis; the present study examined the same parameter categories, though it did not detect a statistically robust difference by activity level for either. Wadsworth et al. [66] examined whether lifestyle factors, including physical activity level, sleep quality, and nutrition, moderate the relationship between mood and gait characteristics in healthy young adults, finding that physical activity was among the factors influencing gait rhythm outcomes; this line of work may offer a useful starting point for future, adequately powered studies designed specifically to test whether physical activity level is associated with temporal gait characteristics in young, healthy populations.

As noted above, skeletal muscle mass was similarly unrelated to lateral speed asymmetry measured via the parkour test (*ρ* = 0.018; 95% CI (−0.32, 0.35); *p* = 0.920; Table 8). This is consistent with D’Hondt et al. [67], who observed morphological lean-mass asymmetry between the left and right leg in young individuals that was not necessarily reflected in functional task performance, supporting the interpretation that muscle-mass and functional asymmetry measures capture largely independent aspects of limb characteristics.

Finally, it was examined whether the absence of association between skeletal muscle mass and physical activity with movement biomechanics also extended to sedentary behavior, i.e., time spent in a sitting position. Spearman correlation analysis found no statistically significant association between sedentary time and any examined gait, asymmetry, or explosive strength parameters (all 95% confidence intervals crossed zero; Table 9). A mild positive association was found between sedentary time and temporal asymmetry, although it was not statistically significant. Time spent seated may be insufficient to produce visible functional changes in a healthy young population, particularly if participants spend adequate time being physically active. Differences in individual patterns of physical activity and everyday movement may also influence the relationship between sitting time and the analyzed parameters. These results are consistent with Sa-nguanmoo et al. [68], who also found no significant differences in gait speed by sedentary behavior. Similar results suggest that other factors influence gait speed. These results can also be compared with a 2016 study. McVeigh et al. [69] observed that young adults spend a significant portion of their time sedentary, while simultaneously being moderately to highly physically active. Prolonged sitting should not be interpreted as equivalent to physical inactivity, since highly active individuals may simultaneously accumulate substantial sitting time—a pattern sometimes described as the ‘active couch potato’ phenomenon [70]. This coexistence of high physical activity and considerable sedentary time has also been documented specifically in university student populations; Moulin et al. [71], in a systematic review of sedentary time among undergraduate students, similarly reported that substantial daily sitting time is common in this population regardless of overall physical activity level. These results may help explain the absence of a significant association between sedentary time and gait, explosive strength, and speed parameters in this study, since time spent sitting does not necessarily indicate low physical activity.

The results contribute to understanding the relationships between sedentary behavior, physical activity, skeletal muscle mass, and movement biomechanics in a healthy young population, a group that, to date, has been studied far less than older and clinical populations. The findings indicate that, in this population, the unadjusted association between skeletal muscle mass and indicators of explosive strength did not remain statistically significant once sex was accounted for, suggesting that this relationship is largely attributable to shared sex differences rather than an independent effect of skeletal muscle mass itself; a similar pattern of caution applies to physical activity level, which showed no robust association with gait biomechanical parameters or gait asymmetry once appropriate statistical scrutiny was applied. Taken together, these findings underscore the importance of accounting for sex and multiple-testing burden when interpreting associations among body composition, physical activity, and movement biomechanics in young, healthy populations. This approach, which combines questionnaire-based assessment, objective body composition analysis, and instrumented biomechanical analysis within the same group of participants, may serve as a foundation for future research in larger, sex-balanced, and adequately powered samples to further confirm or refute these preliminary findings.

### Limitations of the Study

When assessing the results, several methodological limitations must be considered. The cross-sectional design prevents causal conclusions about the direction of the observed relationships. The use of the IPAQ, which is a standard and commonly used tool, depends on participant self-report, which might bring some degree of bias in evaluating physical activity level and sedentary behavior; relatedly, physical activity categories were assigned using a simplified approach based on total weekly MET-minutes rather than the full official IPAQ algorithm, which additionally requires day-level activity patterns. The sample was also unevenly distributed by sex, with a considerably higher proportion of women than men, which may limit the generalizability of the findings; this imbalance is a particular concern for the associations between skeletal muscle mass and explosive strength, which did not remain statistically significant after adjustment for sex, indicating that sex is an important confounding factor that must be accounted for in future research on this topic. The nominal difference in sitting time by sex, which did not remain significant after FDR correction, should likewise be interpreted with caution, as complete sitting-time data were available for only 8 men and 25 women. The asymmetry indices (SAI, TAI, single-leg CMJ asymmetry, and parkour asymmetry) were computed using the manufacturer’s proprietary algorithm, which is not publicly disclosed; different asymmetry formulas can yield substantially different values, and to the authors’ knowledge, no independently published reliability or validity data are currently available for the parkour test used to assess speed and lateral asymmetry in this study. Pre-measurement conditions for bioelectrical impedance analysis (hydration, recent food intake, and alcohol consumption) were based on participant self-report rather than objective verification. Future research could better confirm these results in larger, more varied, and sex-balanced samples, adjusting for additional potential confounders such as body size and training status, and using a mix of subjective and objective methods to measure physical activity.

## 5. Conclusions

The present study primarily examined the relationship between skeletal muscle mass and explosive strength in a healthy, young population, while secondarily exploring its association with sedentary behavior, physical activity, and gait biomechanical parameters. Percentage of skeletal muscle mass was positively associated with lower-limb explosive strength, including countermovement jump height and power and plyometric jump height; however, these associations were not statistically significant after adjustment for sex, suggesting they largely reflect sex differences in both skeletal muscle mass and explosive strength rather than an independent relationship. No association was found between skeletal muscle mass and indicators of lateral asymmetry, either before or after adjustment. Percentage of skeletal muscle mass was not associated with total IPAQ score and showed only a nominal association with IPAQ physical activity category that did not survive correction for multiple testing; this divergence warrants further investigation. Physical activity level was not associated with gait speed, distance, or any other gait biomechanical parameter; neither temporal nor spatial gait asymmetry differed significantly across IPAQ activity categories, although a descriptive pattern of greater temporal asymmetry in the low-activity subgroup should be regarded as exploratory only, given its very small size (*N* = 2). Sedentary behavior was not associated with any gait, explosive strength, or speed parameter, although men reported more sitting time than women, a difference that did not remain significant after FDR correction. Taken together, these findings indicate that, in healthy young adults, associations between body composition, physical activity, and movement biomechanics are more limited and more strongly confounded by sex than unadjusted analyses alone would suggest. Given the cross-sectional design, reliance on a self-reported activity measure, and the sample’s uneven sex distribution, interpret these associations as preliminary. Combining questionnaire-based, body-composition, and instrumented biomechanical assessment within the same young, healthy sample offers a feasible framework for future studies with larger, sex-balanced, and adequately powered populations to confirm and extend these findings.

## Figures and Tables

**Figure 1:**
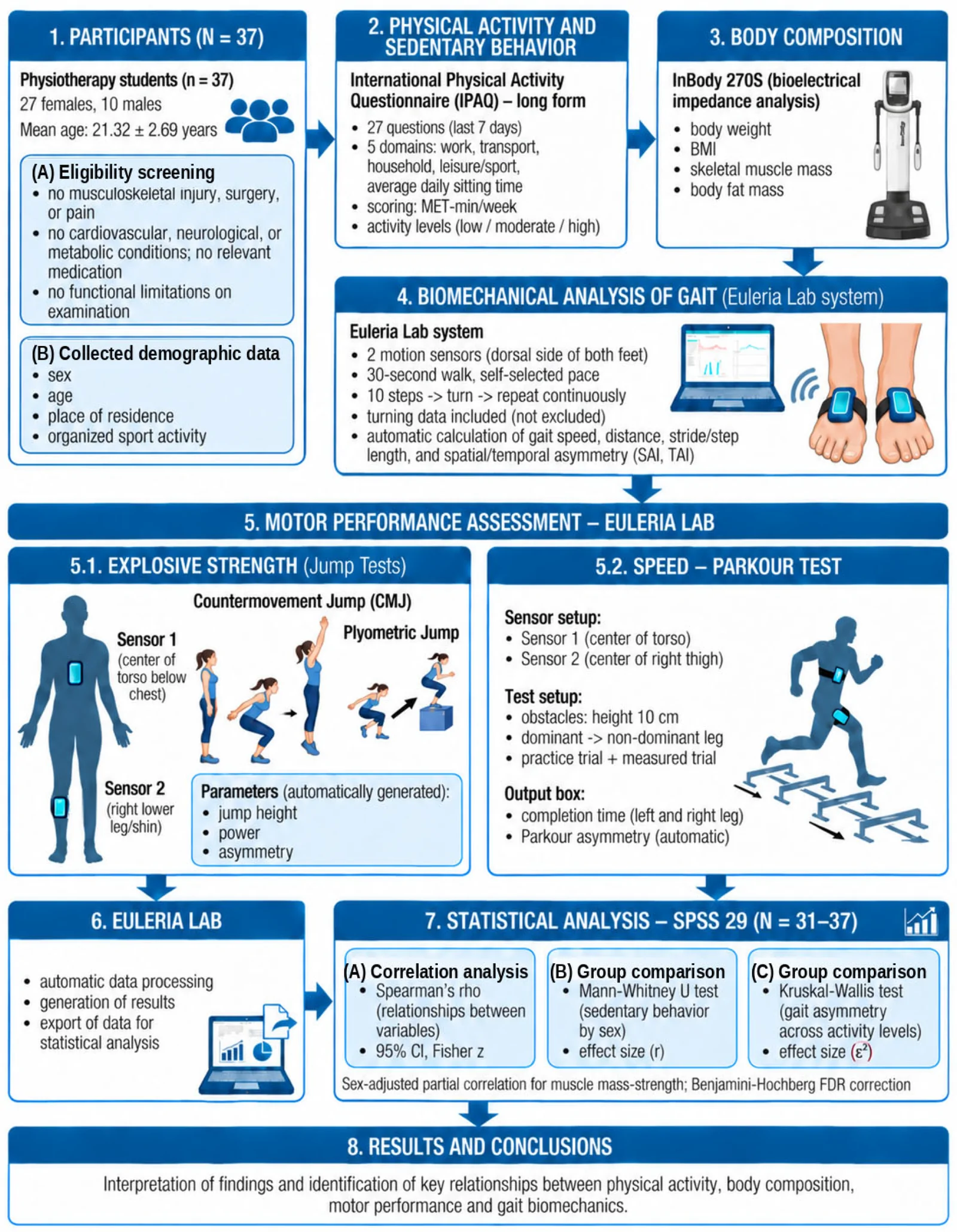
Flowchart of the study protocol.

**Table 1:** Sociodemographic characteristics and physical activity level of the sample (*N* = 37).

Items		*N* (%)
Sex	Female	27 (73.0)
	Male	10 (27.0)
Place of residence	Urban	22 (59.5)
	Rural	15 (40.5)
Participation in organized sports activity	No	21 (56.8)
	Yes, recreational	11 (29.7)
	Yes, competitive	5 (13.5)
IPAQ physical activity category	Low	2 (5.4)
	Moderate	6 (16.2)
	High	29 (78.4)

*N*—number of participants; %—percentage of the sample.

**Table 2:** Differences in sedentary behavior by sex.

Sex	*N*	*M*	Median (IQR)	*SD*	*p* *	*p* (FDR)	*r* (95% CI)
Male	8	7.16	7.71 (5.50–8.79)	2.01	0.034	0.192	0.37 (0.07-0.64)
Female	25	5.36	5.00 (3.86–6.25)	2.12			

*N*—number of participants; *M*—mean; Median (IQR)—median with interquartile range (Q1–Q3) in parentheses; *SD*—standard deviation; *p* (FDR)—*p*-value adjusted using the Benjamini–Hochberg false discovery rate procedure; *r*—effect size (0.1 = small, 0.3 = medium, 0.5 = large) with 95% confidence interval in parentheses. *p* < 0.05; * Mann–Whitney *U* test.

**Table 3:** Descriptive statistics of anthropometric, physical activity, sedentary behavior and biomechanical variables.

Variable	*N*	*M*	*SD*	Min	Max
Age	37	21.32	2.69	18.00	31.00
Height (cm)	37	171.68	10.07	153.00	195.00
Body mass (kg)	37	72.18	16.47	49.00	114.40
Body Mass Index (BMI) (kg/m^2^)	37	24.39	4.75	17.30	37.34
Skeletal muscle mass (%)	37	29.10	8.19	15.80	46.40
IPAQ total (MET-min/week) *	37	7761.06	6167.59	148.50	25,506.00
Sitting (h/day)	33	5.80	2.21	2.14	11.43
Gait speed (m/s)	37	1.10	0.17	0.73	1.69
Gait distance (m)	37	30.97	9.22	20.72	79.74
Spatial asymmetry index (SAI) (%)	37	10.49	14.16	0.40	84.86
Temporal asymmetry index (TAI) (%)	37	3.15	3.70	0.05	15.73
CMJ height (cm)	35	30.57	5.93	18.20	43.20
CMJ power (W/kg)	36	42.44	5.30	29.90	52.00
Single-leg CMJ asymmetry (%)	34	6.10	10.81	0.00	53.00
Plyometric jump—height (cm)	36	29.26	5.95	19.80	41.50
Parkour asymmetry (%)	35	9.42	10.27	0.00	39.00

*N*—number of participants; *M*—mean; *SD*—standard deviation; Min—minimum value; Max—maximum value; MET—Metabolic Equivalent of Task; CMJ—Countermovement Jump; * Median = 6850.00, IQR = 7220.00 (Q1 = 3249.00; Q3 = 10,469.00).

**Table 4:** Correlation between skeletal muscle mass percentage and physical activity level (IPAQ).

Predictor	Outcome	*N*	Spearman *ρ*	95% CI	*p*	*p* (FDR)
Skeletal muscle mass (%)	IPAQ total (MET-min/week)	37	0.224	−0.11, 0.51	0.183	0.430
Skeletal muscle mass (%)	IPAQ physical activity category	37	0.339	0.02, 0.60	0.040	0.192

*N*—number of participants; Spearman *ρ*—Spearman correlation coefficient; 95% CI—95% confidence interval for *ρ*; *p* (FDR)—*p*-value adjusted using the Benjamini–Hochberg false discovery rate procedure; *p* < 0.05; MET—Metabolic Equivalent of Task.

**Table 5:** Correlation between IPAQ physical activity level and gait parameters.

Predictor	Outcome	*N*	Spearman *ρ*	95% CI	*p*	*p* (FDR)
IPAQ total (MET-min/week)	Gait speed (m/s)	37	−0.093	−0.40, 0.24	0.583	0.847
	Gait distance (m)	37	−0.143	−0.45, 0.19	0.399	0.737
	Normalized gait speed (BH/s)	37	−0.071	−0.39, 0.26	0.678	0.847
	Stride L (m)	37	−0.217	−0.51, 0.12	0.197	0.430
	Stride R (m)	37	−0.236	−0.52, 0.10	0.160	0.430
	Step L (m)	37	−0.118	−0.43, 0.21	0.487	0.835
	Step R (m)	37	−0.098	−0.41, 0.23	0.563	0.847
	Spatial asymmetry index (SAI) (%)	37	−0.066	−0.38, 0.26	0.696	0.847
	Temporal asymmetry index (TAI) (%)	37	0.219	−0.11, 0.51	0.192	0.430

*N*—number of participants; Spearman *ρ*—Spearman correlation coefficient; 95% CI—95% confidence interval for *ρ*; *p* (FDR)—*p*-value adjusted using the Benjamini–Hochberg false discovery rate procedure; BH/s—Body heights per second; L—left; R—right; *p* < 0.05.

**Table 6:** Association between percentage of skeletal muscle mass and indicators of explosive strength and lateral asymmetry, unadjusted and adjusted for sex.

Predictor	Outcome	*N*	Unadj. Spearman *ρ*	95% CI	Unadj. *p*	*p* (FDR)	Partial Spearman *ρ* ᵃ	95% CI	Partial *p*
Skeletal muscle mass (%)	CMJ height (cm)	35	0.481	0.18, 0.70	0.003	0.024	0.092	−0.25, 0.42	0.600
	CMJ power (W/kg)	36	0.569	0.30, 0.76	<0.001	0.012	0.307	−0.03, 0.58	0.068
	Single-leg CMJ asymmetry (%)	34	0.059	−0.29, 0.39	0.741	0.847	−0.091	−0.42, 0.26	0.609
	Plyometric jump—height (cm)	36	0.503	0.21, 0.71	0.002	0.024	0.040	−0.30, 0.37	0.817
	Parkour asymmetry (%)	35	0.018	−0.32, 0.35	0.920	0.983	−0.081	−0.41, 0.26	0.638

*N*—number of participants; Unadj. Spearman *ρ*—unadjusted Spearman correlation coefficient; Partial Spearman *ρ*—Spearman partial correlation coefficient; 95% CI—95% confidence interval for *ρ*; *p* (FDR)—unadjusted *p*-value corrected using the Benjamini–Hochberg false discovery rate procedure; CMJ—Countermovement Jump. ᵃ Adjusted for sex.

**Table 7:** Spatial and temporal gait asymmetry by IPAQ category.

Variable	IPAQ Category	*N*	*M*	*SD*	*p* *	*p* (FDR)	ε^2^
Spatial asymmetry index (SAI) (%)	Low	2	7.33	2.10	0.983		−0.058
	Moderate	6	7.19	4.36		0.983	
	High	29	11.40	15.82			
Temporal asymmetry index (TAI) (%)	Low	2	9.90	8.24	0.072		0.096
	Moderate	6	1.32	1.35		0.288	
	High	29	3.06	3.29			

*N*—number of participants; *M*—mean; *SD*—standard deviation; *p* < 0.05; *p* (FDR)—*p*-value adjusted using the Benjamini–Hochberg false discovery rate procedure; ε^2^—epsilon-squared effect size (small ≈ 0.01, medium ≈ 0.06, large ≈ 0.14); a negative value indicates less between-group variation than expected under the null distribution. * Kruskal–Wallis test.

**Table 8:** Association between percentage of skeletal muscle mass and lateral speed asymmetry between the left and right leg.

Predictor	Outcome	*N*	Spearman *ρ*	95% CI	*p*
Skeletal muscle mass (%)	Lateral speed asymmetry (%)	35	0.018	−0.32, 0.35	0.920

*N*—number of participants; Spearman *ρ*—Spearman correlation coefficient; 95% CI—95% confidence interval for *ρ*; *p* < 0.05.

**Table 9:** Association between sedentary behavior and gait, strength, and speed parameters.

Predictor	Outcome	*N*	Spearman *ρ*	95% CI	*p*	*p* (FDR)
Sitting (h/day)	Gait speed (m/s)	33	−0.232	−0.53, 0.12	0.194	0.430
	Spatial asymmetry index (SAI) (%)	33	0.062	−0.29, 0.40	0.732	0.847
	Temporal asymmetry index (TAI) (%)	33	0.214	−0.14, 0.52	0.231	0.462
	CMJ height (cm)	31	0.066	−0.30, 0.41	0.724	0.847
	CMJ power (W/kg)	32	0.011	−0.34, 0.36	0.954	0.983

*N*—number of participants; Spearman *ρ*—Spearman correlation coefficient; *p* < 0.05; 95% CI—95% confidence interval for *ρ*; *p* (FDR)—*p*-value adjusted using the Benjamini–Hochberg false discovery rate procedure; CMJ—Countermovement Jump.

## Data Availability

The data presented in this study are available on request from the corresponding author.

## Supplementary Materials

The following supporting information can be downloaded at: https://www.mdpi.com/article/10.3390/jfmk11040404/s1, Supplementary Material Figure S1: Parkour Test Protocol.

## Abbreviations

The following abbreviations are used in this manuscript:

BMI	Body Mass Index
CMJ	Countermovement Jump
IPAQ	International Physical Activity Questionnaire
MET	Metabolic Equivalent of Task
SAI	Spatial asymmetry index
TAI	Temporal asymmetry index
