# Supplementary material for: Skeletal Muscle Mass and Explosive Strength in Young, Healthy Adults: Associations with Gait Biomechanics, Speed and Sedentary Behavior—A Cross-Sectional Study
Source: J Funct Morphol Kinesiol. 2026 Oct 3;11(4):404. doi: 10.3390/jfmk11040404 (PMC13692536; doi:10.3390/jfmk11040404)
Supplement: Supplementary file 1 [file jfmk-11-00404-s001.zip › jfmk-4561569-supplementary.pdf]

Parkour Test Protocol

The Parkour test was used to assess the speed of performing a specified motor task separately with the left and right leg. The test was administered following the standard protocol provided by the manufacturer (Euleria Lab, Euleria Health, Italy) for use with this sensor system. The course consisted of a central longitudinal line along which four transverse hurdles were placed, arranged alternately on the left and right sides of the central line. The height of each hurdle was 10 cm. Two Euleria Lab system sensors were used to record movement. Sensor 1 was placed at the mid-trunk, while Sensor 2 was placed at the mid-right thigh. The position of Sensor 2 was not changed between testing of the left and right leg. After sensor placement and activation, the system was recalibrated. The test was performed separately with the left and right leg, with the dominant leg tested first, followed by the non-dominant leg. Before the measured trial for each leg, participants performed one practice trial that was not included in the final result; this practice trial served to familiarize participants with the hurdle arrangement and task execution. During the measured trial, starting from the starting position of the course, participants crossed over each hurdle three times with the selected leg, after which they moved to the next section of the course. The task was performed continuously along the entire course, with instructions to complete it in the shortest possible time while maintaining correct movement execution. After completing measurement with one leg, the same procedure was repeated with the other leg (Figure S1.). To the authors' knowledge, no independently published reliability or validity data are currently available for this test; this is acknowledged as a limitation in the main manuscript.

Completion time for the entire course was recorded in seconds, separately for the left and right leg, with a shorter time indicating faster performance. Based on these completion times, the Euleria Lab device automatically generated an indicator of speed asymmetry between the left and right leg ("Parkour asymmetry"), which was the parkour outcome analyzed in this study. This indicator was taken directly from the system report and was not additionally calculated by the researchers; the manufacturer's computational algorithm is proprietary and not publicly disclosed.

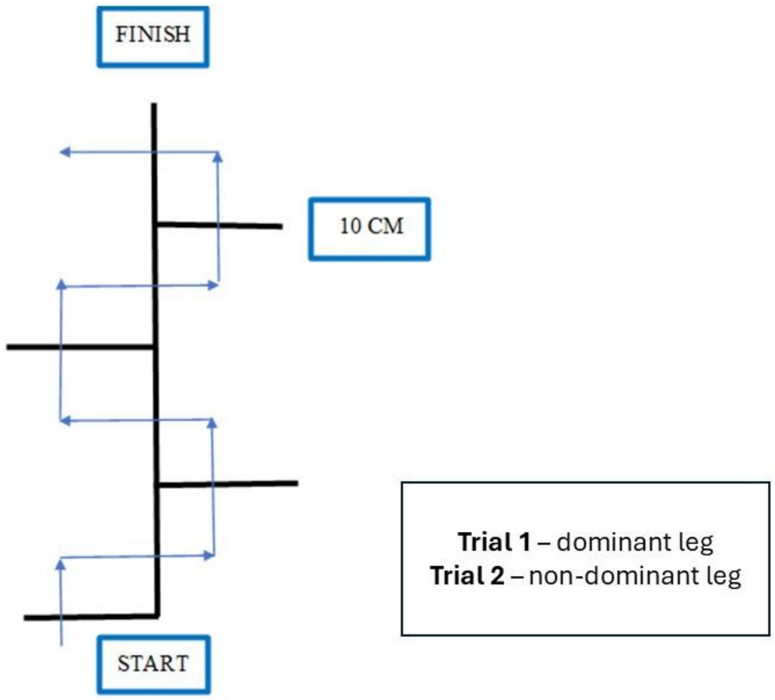

Figure S1. Parkour test protocol
